# Association of gut microbiota and SCFAs with finishing weight of Diannan small ear pigs

**DOI:** 10.3389/fmicb.2023.1117965

**Published:** 2023-01-27

**Authors:** Qun Lan, Yuju Lian, Peiya Peng, Long Yang, Heng Zhao, Peng Huang, Haiming Ma, Hongjiang Wei, Yulong Yin, Mei Liu

**Affiliations:** ^1^College of Animal Science and Technology, Hunan Agricultural University, Changsha, China; ^2^State Key Laboratory for Conservation and Utilization of Bio-Resources in Yunnan, Yunnan Agricultural University, Kunming, China; ^3^Key Laboratory of Agro-ecological Processes in Subtropical Region, Institute of Subtropical Agriculture, Chinese Academy of Sciences, Hunan Provincial Engineering Research Center for Healthy Livestock and Poultry Production, Scientific Observing and Experimental Station of Animal Nutrition and Feed Science in South-Central, Ministry of Agriculture, Changsha, China; ^4^Kunpeng Institute of Modern Agriculture at Foshan, Foshan, China

**Keywords:** Diannan small-ear pigs, finishing weight, 16S rRNA gene, metagenomic, SCFAs, gut microbiome

## Abstract

Finishing weight is a key economic trait in the domestic pig industry. Evidence has linked the gut microbiota and SCFAs to health and production performance in pigs. Nevertheless, for Diannan small ear (DSE) pigs, a specific pig breed in China, the potential effect of gut microbiota and SCFAs on their finishing weight remains unclear. Herein, based on the data of the 16S ribosomal RNA gene and metagenomic sequencing analysis, we found that 13 OTUs could be potential biomarkers and 19 microbial species were associated with finishing weight. Among these, carbohydrate-decomposing bacteria of the families *Streptococcaceae*, *Lactobacillaceae*, and *Prevotellaceae* were positively related to finishing weight, whereas the microbial taxa associated with intestinal inflammation and damage exhibited opposite effects. In addition, interactions of these microbial species were found to be linked with finishing weight for the first time. Gut microbial functional annotation analysis indicated that CAZymes, such as glucosidase and glucanase could significantly affect finishing weight, given their roles in increasing nutrient absorption efficiency. Kyoto Encyclopedia of Genes and Genomes (KEGG) Orthologies (KOs) and KEGG pathways analysis indicated that glycolysis/gluconeogenesis, phosphotransferase system (PTS), secondary bile acid biosynthesis, ABC transporters, sulfur metabolism, and one carbon pool by folate could act as key factors in regulating finishing weight. Additionally, SCFA levels, especially acetate and butyrate, had pivotal impacts on finishing weight. Finishing weight-associated species *Prevotella* sp. *RS2*, *Ruminococcus* sp. *AF31-14BH* and *Lactobacillus pontis* showed positive associations with butyrate concentration, and *Paraprevotella xylaniphila* and *Bacteroides* sp. *OF04*-*15BH* were positively related to acetate level. Taken together, our study provides essential knowledge for manipulating gut microbiomes to improve finishing weight. The underlying mechanisms of how gut microbiome and SCFAs modulate pigs’ finishing weight required further elucidation.

## Introduction

Domesticated pigs account for a high proportion of meat production in the world and also serve as an important animal model for basic biomedical research studies (Lunney, 2007). The swine intestinal tract harbors trillions of diverse microorganisms, which get involved in regulating host nutrient digestion, energy absorption, and disease development (Zmora et al., 2019). Short-chain fatty acids (SCFAs), mainly comprised of propionate, butyrate, and acetate, are regarded as important metabolites derived from bacteria-dependent hydrolysis of indigestible dietary fibers, which play crucial roles in energy metabolism modulation and improving intestinal homeostasis (Priyadarshini et al., 2018).

Recently, emerging evidence suggests that SCFAs and gut microbiomes exert positive effects in regulating the growth performances of livestock animals. For instance, the increased abundance of bacteria belonging to families *Ruminococcaceae* and *Lachnospiraceae* could generate a high concentration of butyrate, which is responsible for the higher feed intake and body weight of broilers (Yacoubi et al., 2018). The genus *Prevotella*, genus *Faecalibacterium*, and family *Ruminococcaceae* are participated in digesting dietary fibers which subsequently influence SCFA production, which played vital roles in modulating body weight and feed intake of pigs (Wang J. et al., 2020). Moreover, research pointed out that the significant improvement in feed intake and average daily gain (ADG) of fattening cattle were closely related to levels of acetic acid and propionic acid, and their producing-bacteria *Acetobacterium* and *Fournierella*, respectively (Zhuang et al., 2021). Additionally, the increased abundance of *Bifidobacterium* and *Lactobacillus* in the gut of broiler chickens has contributed to the generation of lactate and SCFAs, which possess positive effects on ADG and food conservation rate (Yu et al., 2019).

Finishing weight is considered to be a critical growth trait of pigs, which directly reflects meat production. Nevertheless, only few works have unraveled the potential relationship between the intestinal microbiome and the finishing weight of pigs. For example, research indicated that the abundance of *Parabacteroides*, *Bacteroides*, and *ClostridiumXI* showed positive associations with the final body weight of pigs (Yang et al., 2021b). The body weight of Enshi pigs at the finishing stage was positively correlated with *Ruminococcaceae UCG-005* and *Alloprevotella* (Tang et al., 2020). In addition, genera *Prevotella*, unclassified *Bacteroidales*, and unclassified *Veillonellaceae* in multi-intestinal segments of pigs were positively associated with finishing weight (De Rodas et al., 2018). In contrast, genera *Stenotrophomonas*, *Sphaerochaeta*, and *Desulfovibrio* in the gut microbial communities of pigs were negatively associated with finishing weight (Tang et al., 2020). Another study reported that genera *Parabacteroides*, *Desulfovibrio*, and *Treponema* had negative correlations with the body weight of pigs at the finisher stage (Oh et al., 2020). Moreover, the final body weight of the three-way hybrid pig was negatively correlated with the genera *Roseburia*, *Prevotella*, and *Anaerovibrio* (Yang et al., 2021b). However, these above-mentioned studies are mainly based on 16S rRNA gene sequencing analysis, which cannot identify the specific species and functional capacities that affect finishing weight. As a specific pig breed of China, the Diannan small-ear (DSE) pigs are primarily raised in the southern region of Yunnan province with a subtropical climate, and are characterized by more fat deposition, lower growth rate, better meat quality, and strong resistance to adverse surroundings (Wang et al., 2015). The potential characters of the gut microbiome and SCFAs in regulating the finishing weight of DSE pigs remain largely unknown. Hence, we devised the present study aimed to investigate the effects of gut microbiome and SCFAs on body weight of DSE pigs in finishing phase using data of 16S rRNA gene sequencing, metagenomic sequencing, and fecal SCFAs. We finally identified some microbial species and metabolic functions related to finishing weight. Also, the correlations between SCFAs concentration and finishing weight were unraveled. Our study helps to uncover the effects of gut microbiome and SCFAs on modulating finishing weight and hence provide certain reference significances for improving production performance of DSE pigs and others.

## Materials and methods

### Experimental pigs and sample collection

One hundred and two Diannan small-ear (DSE) pigs (40 ± 3 days, 48 females and 54 males) with similar weaning weights were grouped into different commercial pens and bred under the same nutritional and management conditions until the finishing stage (345 ± 3 days). At the finishing stage, all DSE pigs were fed with same diet two times 1 day using the compound feeds containing 14–16% of crude protein, 0.25–0.60% of sodium chloride, 0.60–1.50% of calcium, 8% of coarse fiber, 3,100–3,200 kJ of digestible energy, and 0.70% of lysine, and offered an *ad libitum* water. The finishing body weight of all DSE pigs was measured and recorded. Then, finishing weights were sorted to select the bottom six pigs with the lowest body weight (low group) and the top six pigs with the highest body weight (high group) for both 16S rRNA gene sequencing and metagenomic sequencing. The normal distribution of finishing weight for all DSE pigs was displayed in Supplementary Figure S1A. There exists a significant difference in finishing weight among low and high groups (Supplementary Figure S1B, FDR adjusted *p* < 0.01). All DSE pigs were healthy and not fed with probiotics, prebiotics, antibiotics, and anti-coccidial drugs throughout the whole experimental period. The fresh feces of the DSE population were collected directly from the rectum. All samples were stored in the 2-ml sterilized plastic sampling tubes and then dipped into liquid nitrogen immediately. After long-distance transportation, samples were stored in a refrigerator (−80°C) until processing.

### DNA extraction and 16S rRNA gene sequencing

The QIAamp fast DNA stool Mini Kit (QIAGEN, Germany) was selected to extract microbial DNA based on the manufacturer’s guidelines. The concentration and quality of stool DNA were assessed by spectrophotometer Nanodrop 2000 (Thermo Scientific, United States) and 1.5% agarose gel electrophoresis, respectively. The specific primer 338F (5′-ACTCCTACGGGAGGCAGCA-3′) and 806R (5′-GGACTACHVGGGTWTCTAAT-3′) was used to amply the hypervariable regions (V3–V4) for 16S rRNA gene. The PCR amplification was accomplished by following steps: initial 94°C denaturation step for 5 min, subsequently, 30 cycles of 95°C for 30 s, 55°C for 25 s, and 72°C for 25 s followed by a final extension step for 10 min at 72°C. All amplicons were sequenced using the paired-end method on a MiSeq platform (Illumina, United States) following the standard protocols. The quality control process of raw data was performed by using QIIME (v.1.9.1), including removing primers, barcodes, and low-quality sequences (Caporaso et al., 2010). FLASH (v.1.2.11) was used for merging high-quality paired-end reads into tags (Magoc and Salzberg, 2011). Before further analysis, the library size of sequences was rarefied to 40,000 tags for each sample to normalize the sequencing depth. USEARCH (v.11.0) was used for matching tags into operational taxonomic units (OTUs) based on 97% sequence identity (Edgar, 2010). We filtered out those OTUs as following criteria: relative abundance was less than 0.05% and OTUs were presented in 1% of the experimental pigs. OTU taxonomic category assignments were finished by using the SILVA database (v.138.1; Quast et al., 2013). The MOTHUR (v.1.28.1; Chappidi et al., 2019) and QIIME were used for calculating the alpha and beta diversity indices, respectively.

### Biomarker discovery in 16S rRNA data

To identify the biomarker characteristics among low and high finishing weight groups, linear discriminant analysis effect size (LEfSe; Segata et al., 2011) was performed based on a normalized OTU relative abundance matrix. As a tool for high-dimensional biomarker identification and explanation, the LEfSe algorithm uses the non-parametric factoria Kruskal-Wallis test to identify the features with significant differences and performs LDA to estimate the effect size of each feature. The LDA threshold score and significant level in the current study were set at 2 and 0.05, respectively.

### Metagenomic sequencing

Following the instruction of the manufacturer, a paired-end (PE) DNA library was established for each sample, and sequencing was implemented on the Illumina HiSeq 4000 platform. Quality control, adapter trimming, and low-quality read removal of sequenced data were proceeded by software fastp (v.0.23.2; Chen et al., 2018) to achieve clean data, and an average of 59,008,155 high-quality reads was obtained for each sample. The MEGAHIT (v.1.2.9; Li et al., 2015) was then used for assembling high quality reads into contigs, and the length of contigs with more than 200 bp was used to perform open reading frames (ORF) prediction using MetaGeneMark (v.3.25; Zhu et al., 2010). We used Cd-hit (v.4.6.8; Fu et al., 2012) to discard the redundant genes derived from the predicted ORFs for constructing the non-redundant gene catalog. The gene abundance content was obtained by mapping the high-quality reads against the non-redundant gene catalog *via* MOCAT (v2.0; Kultima et al., 2016). The program of hmmscan in HMMER (v.3.0; Eddy, 2011) was used to annotate Carbohydrate-Active enZYmes (CAZymes). Profiles of KEGG pathway annotation information and KEGG Orthologies (KOs) were extracted from the Kyoto Encyclopedia of Genes and Genomes (KEGG) database using GhostKOALA (Kanehisa et al., 2016).

### Determinations of SCFA in fecal samples

The measurement of SCFAs concentrations in stool samples was using gas chromatography on a Shimadzu GC-2010 plus system with a flame ionization detector (Shimadzu, Japan) according to the approaches previously described with small modifications (Xu et al., 2021). A total of 1,000 mg fresh feces sample was vortex-mixed with 5 ml ultrapure water and centrifuged (1,000 rpm) at 4°C for 30 min. Subsequently, the supernatant (900 μl) which was mixed with 100 μl metaphosphoric acid solution (25%; Macklin, United States) for 3 or 4 h was used for filtering by a 0.45 μm filter membrane. Aliquots of the supernatants (600 μl) were pipetted into a glass gas chromatography vial to make preparations for the following GC instrument analysis. Briefly, the temperature of the flame ionization detector was set at 280°C. The carrier gas was highly purified N2 (99.99%) with a flow rate of 0.8 ml/min. The concentration of SCFAs in fecal samples was determined by comparing them with the standard curve. We used Mantel tests based on the Spearman method to perform the correlation analysis between SCFAs levels and finishing weight-associated species.

### Interaction network analysis

To construct the interaction network of all finishing weight associated species, the pair-wised Spearman correlations analysis was performed between these species. Spearman’s correlation coefficient of less than 0.65 and adjust *p* value of more than 0.05 were excluded, which allows us to only focus on those species that strongly co-occurred and were more likely to affect each other. The major modules in the interaction network were calculated and visualized using Gephi software (v.0.9.2; https://gephi.org/). After that, Spearman’s correlation analysis was used to test for the correlations between finishing weight phenotypic value and modules.

### Basic statistical analysis

The differential metagenomic species and function capacities for the gut microbiome between low and high finishing pigs were detected by the Wilcoxon test with FDR correction. Statistical comparisons of SCFAs in stool samples were done using the two-sided Student’s *t*-test (unpaired) with FDR correction between low and high finishing weight pigs. Interactive Tree Of Life (iTOL) was used to visualize the differential metagenomic species, and the other charts were finished by R software.

## Results

### Comparison of bacterial diversity and microbial composition

**Figure 1:**
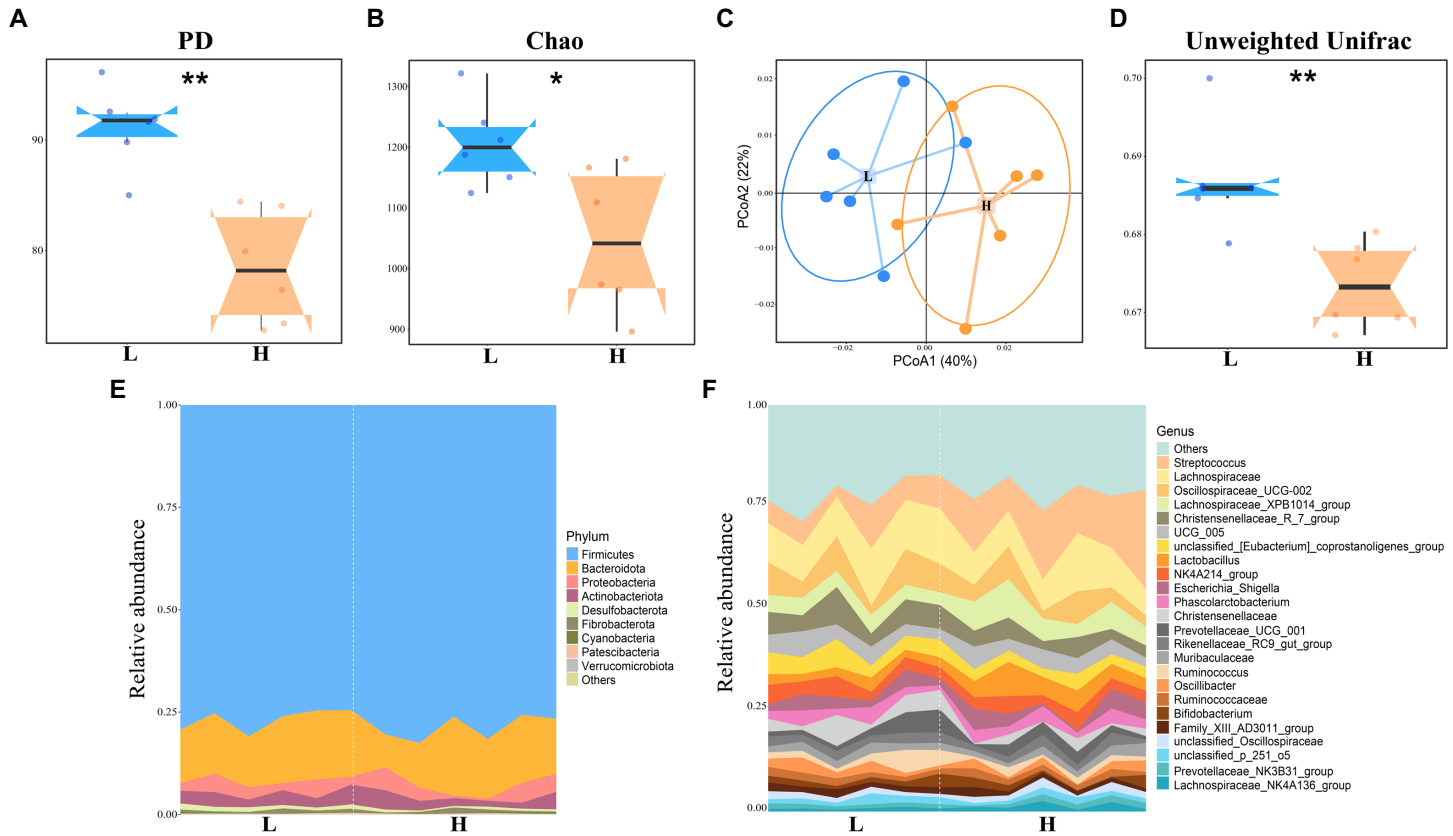
Alpha, beta-diversity comparison, and microbial composition among low and high finishing weight groups. **(A)** Comparison of PD index for two groups. **(B)** Comparison of Chao index between two groups. **(C)** Principal Coordinate Analysis (PCoA) analysis for gut microbial community structures based on Unweighted Unifrac distance. **(D)** Unweighted Unifrac distance metric. **(E,F)** The gut microbial composition for low and high finishing weight DSE pigs in phylum **(E)** and genus **(F)** levels. The box means the interquartile range, and the box with the midline represented the median. “L” and “H” represent low and high finishing weight, respectively; “*” FDR adjusted *p* < 0.05, “**” FDR adjusted *p* < 0.01.

The bacterial diversity (PD and Shannon) and richness (Chao) indices for alpha-diversity were compared to discover the difference for the gut microbial community. As shown in Figure 1A, the PD index in high finishing weight pigs was significantly decreased in comparison with low finishing weight pigs (FDR adjusted *p* < 0.01). In addition, the same tendency was also observed in the Chao index (FDR adjusted *p* < 0.05; Figure 1B). The Shannon index between the two groups did not exhibit a statistical difference while the value in the high finishing weight group was lower than the low finishing weight group (Supplementary Table S1).

Principal Coordinate Analysis (PCoA) was performed to observe the change of gut microbial community structures between two experimental populations. The distribution of microbiota between the two groups was clustered separately along principal coordinates (Figure 1C). The analysis of unweighted UniFrac distance metric comparison indicated that low finishing weight pigs had higher dissimilarity among intestinal microbiota than high finishing weight pigs (Figure 1D, FDR adjusted *p* < 0.01).

For gut microbial composition, *Firmicutes*, *Bacteroidetes,* and *Proteobacteria* were the top three dominant phyla (Figure 1E) and represented more than 95.10% of all sequence reads. At the genus level, *Streptococcus*, *Lachnospiraceae*, *Oscillospiraceae*_*UCG*-*002*, and *Lachnospiraceae*_*XPB1014*_*group* were the first four predominant genera (Figure 1F). Approximately 0.03% of sequences in fecal samples cannot be assigned to a certain rank (unclassified; Supplementary Table S2).

### Differential microbiota compositions in low and high finishing weight groups

**Figure 2:**
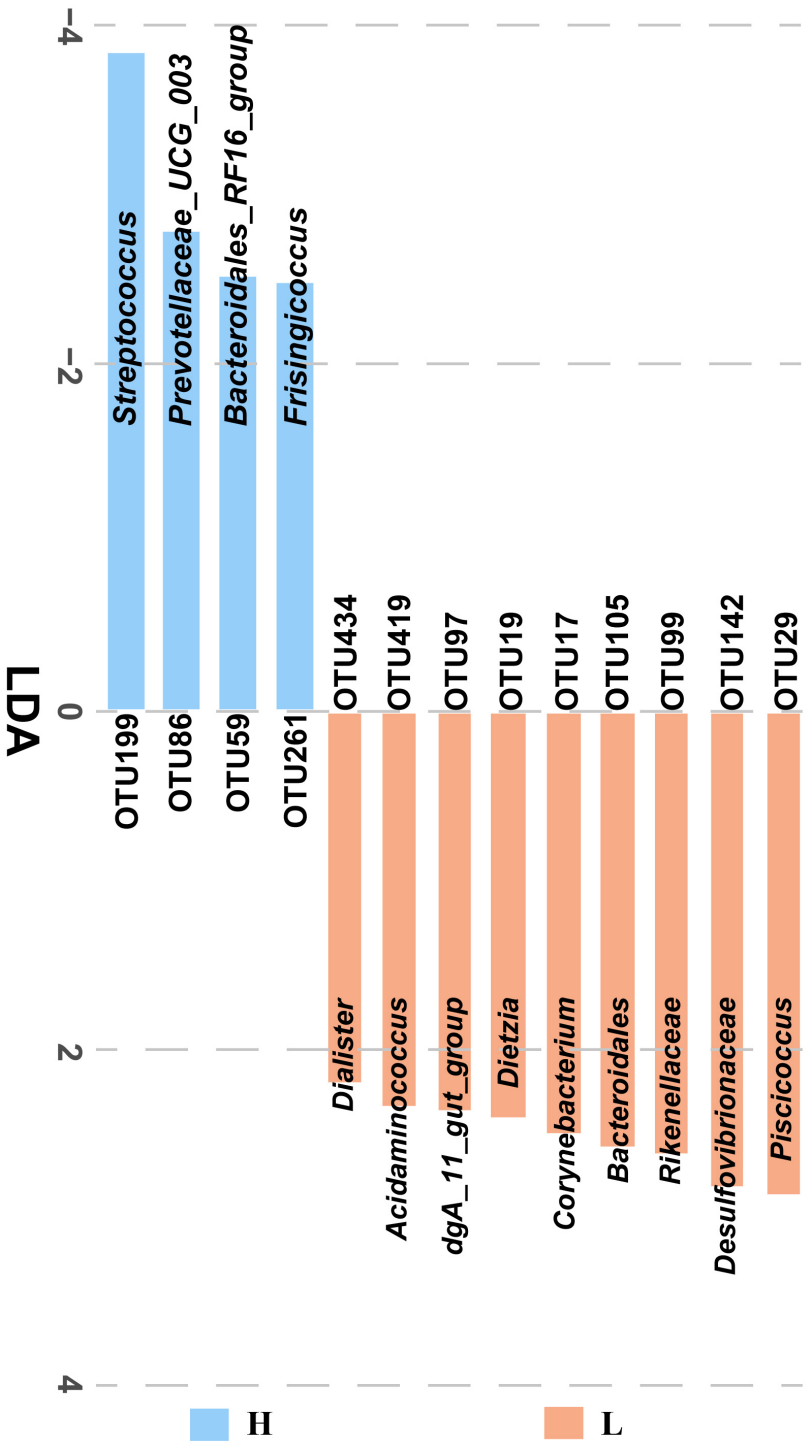
The identified significantly different OTUs in low and high finishing weight pigs. The “H” under the blue bar represents the high finishing weight group, and the “L” under the orange bar represents the low finishing weight group.

The fecal microbiota from low and high finishing weight individuals was analyzed by LEfSe multilevel species discrimination. A total of 13 OTUs were identified (Figure 2). Nine OTUs were specifically found in low finishing weight group, namely, genera *Piscicoccus*, *Corynebacterium*, *Dietzia*, *dgA*_*11*_*gut*_*group*, *Acidaminococcus*, *Dialister*, families *Rikenellaceae* and *Desulfovibrionaceae*, and class *Bacteroidales*. Whereas for the high finishing weight group, three genera *Streptococcus*, *Prevotellaceae UCG*-*003*, *Frisingicoccus,* and one family *Bacteroidales_RF16_group* could be the potential biomarkers.

### Microbial species associated with finishing weight

**Figure 3:**
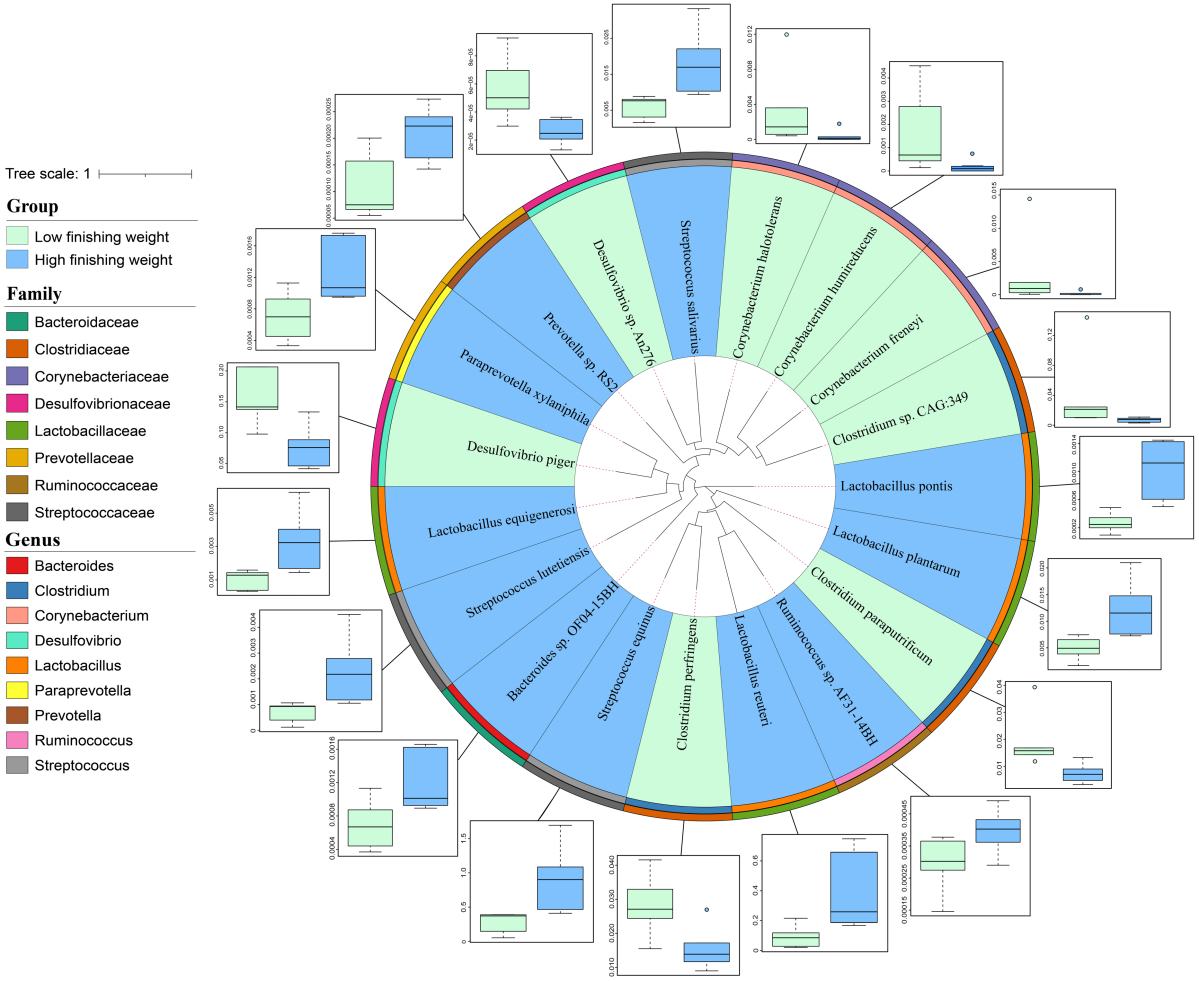
The phylogenetic relationships and abundance comparison of metagenomic species in low and high finishing weight pigs. The phylogenetic tree of species is displayed as the innermost layers. Labels with green and blue colors represent low and high finishing weight groups, respectively. The different color strips in the third and four layers, respectively, correspond to different genera and families as indicated by the color codes on the left. The outermost layer was the boxplot representing the abundance comparison of finishing weight associated species. The dots in the bar chart represent species abundance.

At the species level, 16S rRNA gene sequencing possesses large limitations in both accuracy and resolution. Herein, we performed metagenomic sequencing for 12 individuals to further identify the differential species between low and high finishing weight groups. A total of 19 species showed different enrichments among two groups identified by the Wilcoxon rank-sum test with FDR correction using metagenomic sequencing data (Figure 3 and Supplementary Table S3, FDR adjusted *p* < 0.05). For low finishing weight group, *Desulfovibrio piger* and *Desulfovibrio* sp. *An276* from the family *Desulfovibrionaceae* were the most abundant species. In addition, three species belong to the family *Clostridiaceae*, including *Clostridium perfringens*, *Clostridium* sp. *CAG:349*, and *Clostridium paraputrificum* were abundant in pigs with low finishing weight. Three species from the member of family *Corynebacteriaceae*, named *Corynebacterium freneyi*, *Corynebacterium halotolerans,* and *Corynebacterium humireducens* were also enriched in the low finishing weight group. Conversely, we found that *Streptococcus equinus*, *Streptococcus salivarius*, and *Streptococcus lutetiensis* (members of the family *Streptococcaceae*) were augmented in high finishing weight group, followed by *Lactobacillus reuteri*, *Lactobacillus pontis*, *Lactobacillus plantarum*, and *Lactobacillus equigenerosi*. Moreover, *Prevotella* sp. *RS2*, *Ruminococcus* sp. *AF31-14BH*, *Paraprevotella xylaniphila*, and *Bacteroides* sp. *OF04-15BH* were also abundant in the high finishing weight group.

### Interactions of microbial species associated with finishing weight

**Figure 4:**
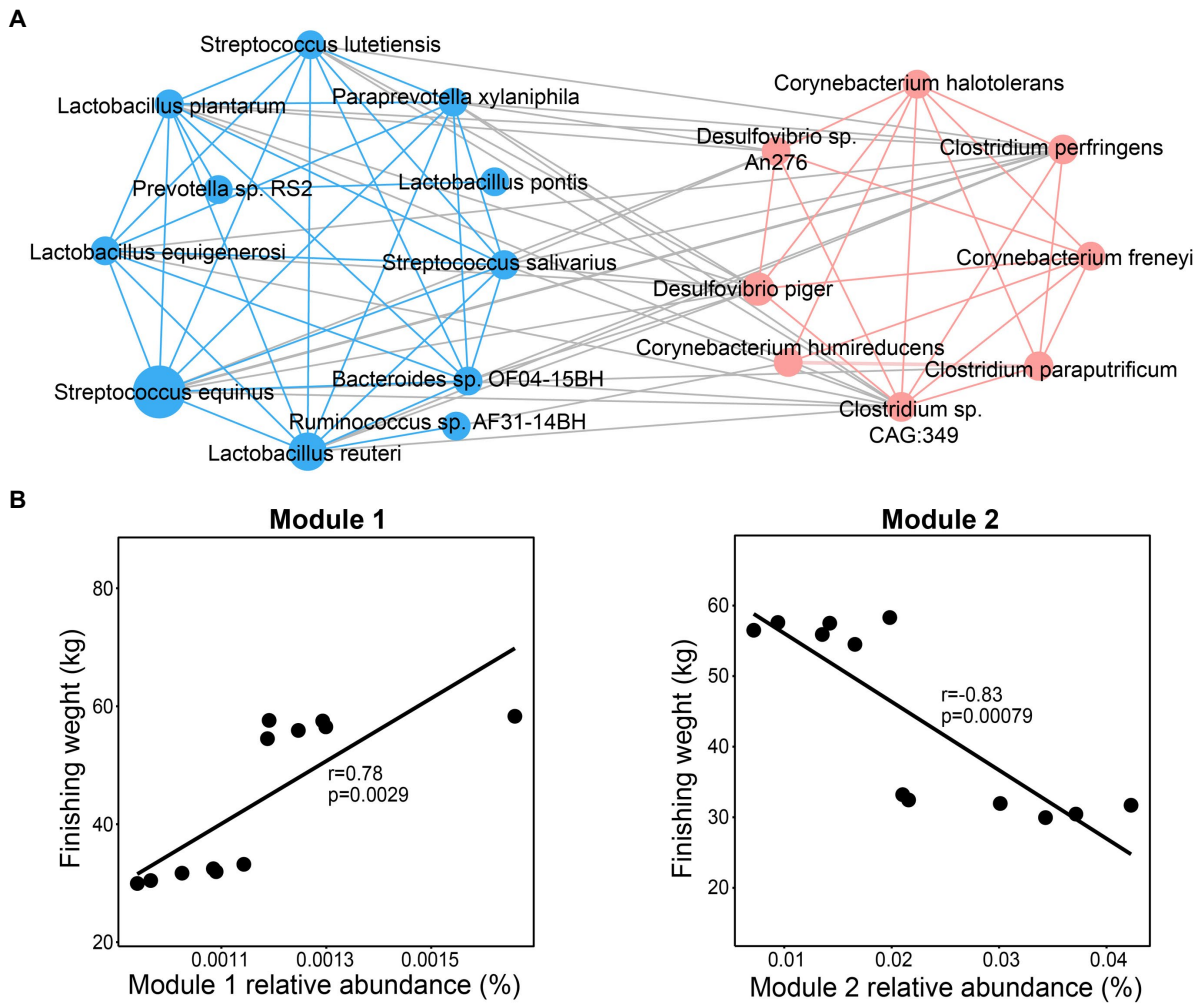
Interactions among the species responding for finishing weight. **(A)** The interaction network among finishing weight-related species. The blue nodes and lines, respectively, denote positively associated species and positive correlations among them. The coral nodes and lines, respectively, represent negatively associated species and positive correlations among them. Lines with gray color indicate a negative correlation. The node size was drawn according to the average relative abundance of different species. Each line linked to nodes represents significant correlations (FDR adjusted *p* < 0.05, |*r*| > 0.65). **(B)** The associations of the abundance of Module 1 and Module 2 with finishing weight.

To determine whether these significantly enriched species play a vital role in affecting the finishing weight by their interactions, they were used to estimate the correlation coefficient using the Spearman method. The interaction network consisted of two modules (Figure 4A). We found that dominant species from family *Desulfovibrionaceae*, *Corynebacteriaceae,* and *Clostridiaceae* were clustered into Module 1 through intra-positive interactions, whereas Module 2 was constituted by intra-positive interactions among the species mainly from families *Streptococcaceae*, *Lactobacillaceae*, and *Prevotellaceae*, which were positively related to finishing weight. In addition, as shown in Figure 4B, we found that Module 1 was positively associated with finishing weight, but Module 2 was negatively correlated with finishing weight.

### Functional compositions of the gut microbiome related to finishing weight

Functionalities of the gut microbiome associated with finishing weight were analyzed by comparing the abundances of KEGG items and CAZymes among low and high finishing individuals.

**Figure 5:**
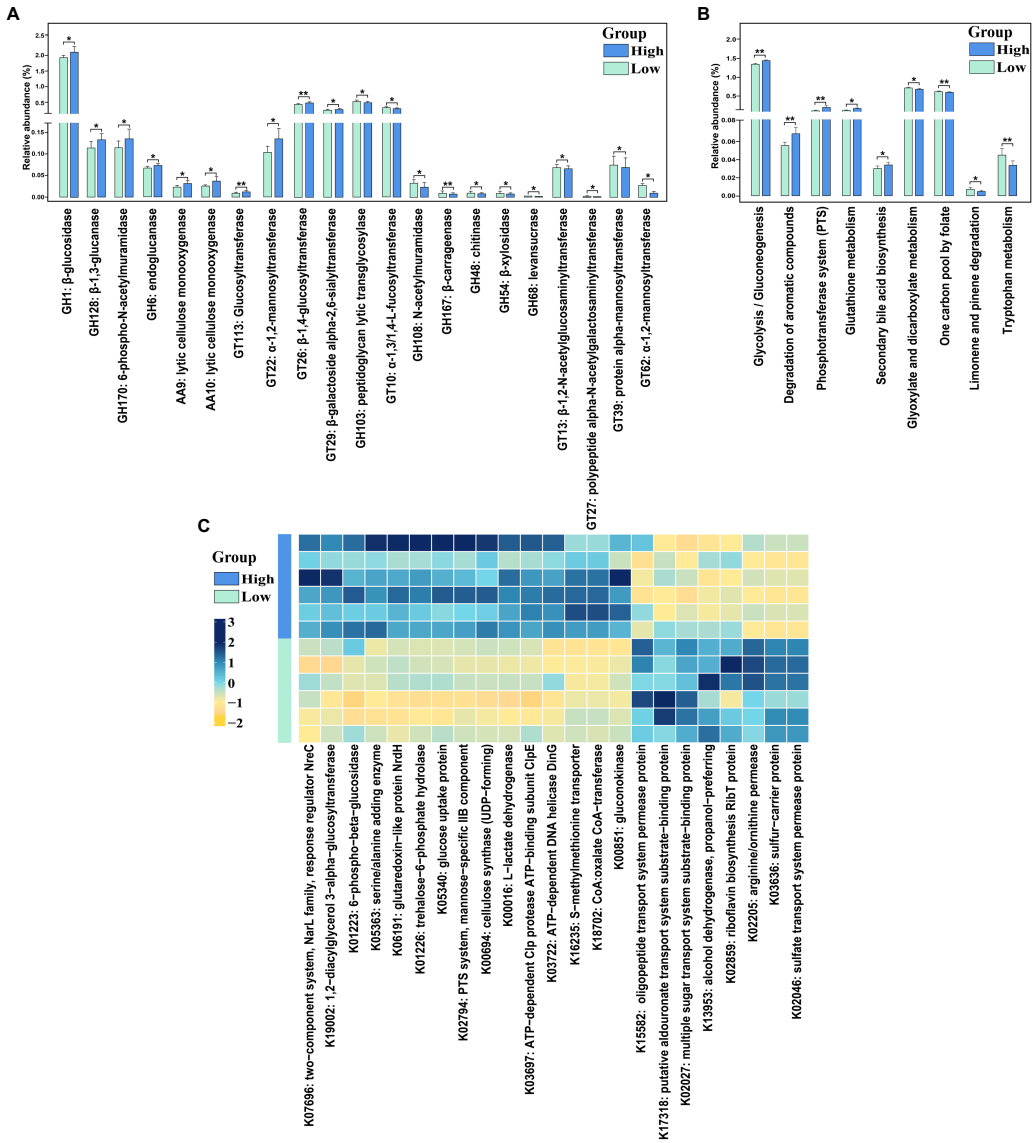
The CAzymes and KEGG function terms exhibiting different enrichments between low and high finishing weight pigs. **(A)** The differential CAzymes are detected to relate with finishing weight. **(B)** The differential KEGG pathways are identified to associate with finishing weight. **(C)** Heat map of KOs displaying different enrichments between low and high finishing weight pigs. “*” and “**” represents for FDR adjusted *p* < 0.05 and *p* < 0.01, respectively. The rectangles with green and blue colors represent low and high finishing weight groups, respectively.

Twenty-one CAZymes with significantly different abundances were detected (Figure 5A, FDR-adjusted *p* < 0.05). Among these, 11 CAZymes especially glucosidase and glucanase (e.g., GH1, GH128, and GH6), lytic cellulose monooxygenase (e.g., AA9 and AA10), and Glucosyltransferase (e.g., GT113 and GT26) were significantly enriched in high finishing weight group. While regarding low finishing weight pigs, we found that mannosyltransferase (e.g., GT39 and GT62), peptidoglycan lytic transglycosylase, and levansucrase had significantly higher abundances.

A total of nine differential enriched KEGG pathways were detected in the gut microbiome of pigs with distinct finishing weights (Figure 5B, FDR-adjusted *p* < 0.05). We found that one carbon pool by folate, limonene, and pinene degradation, and tryptophan metabolism were more active in the gut microbial communities of the low finishing weight group. Meanwhile, in pigs with high finishing weight, gut microbiota was more capable of operating glycolysis/gluconeogenesis, degradation of aromatic compounds, phosphotransferase system (PTS), glutathione metabolism, and secondary bile acid biosynthesis.

On the other hand, 23 KOs showed distinct abundances in pigs with low and high finishing weights (Figure 5C, FDR-adjusted *p* < 0.05). Eight KOs mainly related to ABC transporters (e.g., K15582 and K17318), Fatty acid degradation (e.g., K13953), and Sulfur metabolism (e.g., K02046) were plentiful in low finishing weight group. The other 15 KOs were more abundant in high finishing weight group, most of which were associated with glycolysis/gluconeogenesis (e.g., K00016 and K01223), carbohydrate metabolism (e.g., K01226, K02794, and K00694), lipid metabolism:(e.g., K19002), and the biosynthesis of secondary metabolites (e.g., K00851).

### Changes in fecal SCFAs linked to finishing weight

**Figure 6:**
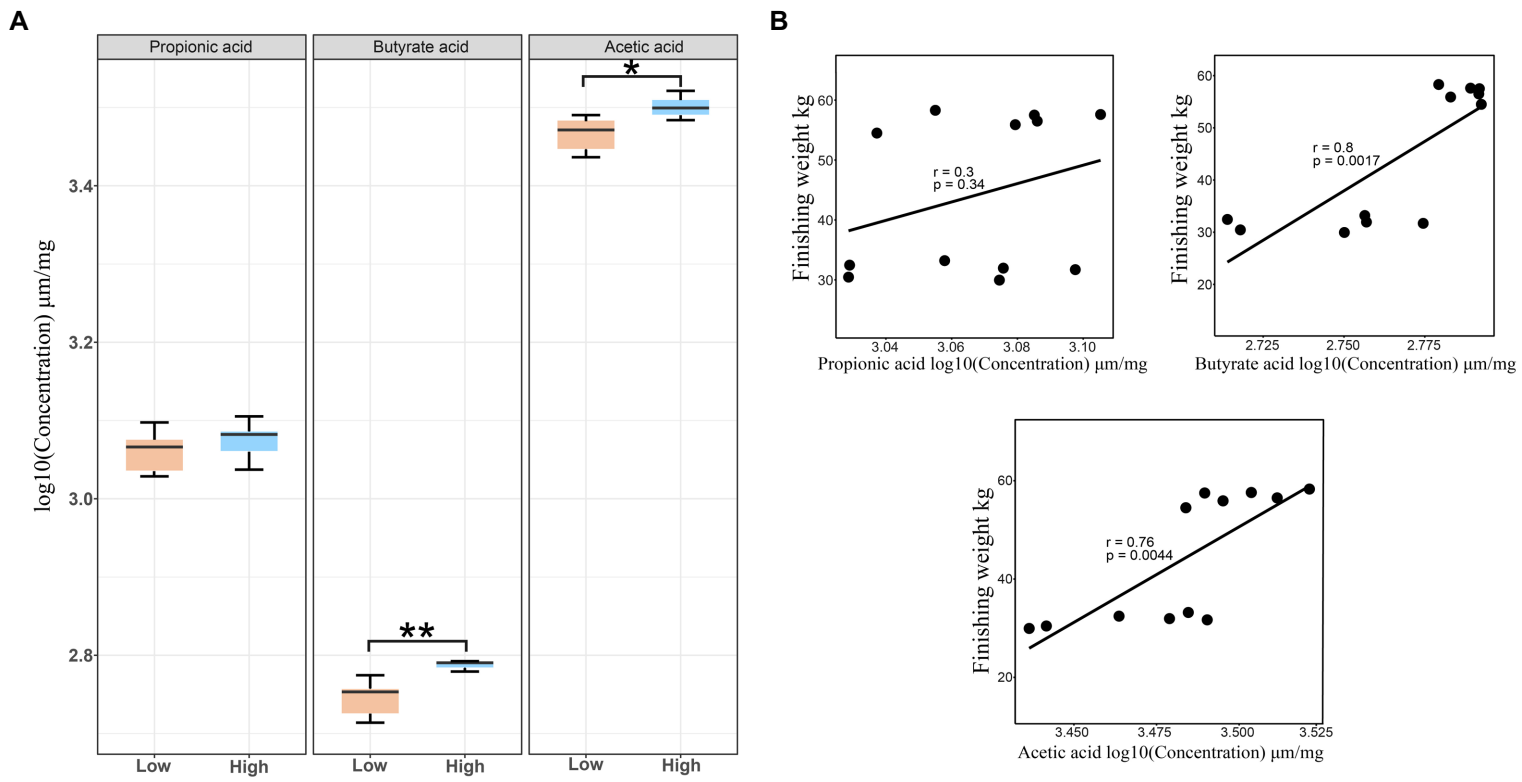
Stool short-chain fatty acid concentrations in low and high finishing weight pigs and their correlations with finishing weight. **(A)** The comparisons of fecal short-chain fatty acid levels between low and high finishing weight pigs. **(B)** The correlations between stool short-chain fatty acid levels and finishing weight. “*” and “**” represents for FDR adjusted *p* < 0.05 and *p* < 0.01, respectively.

Owing to the SCFAs produced by intestinal microbiota playing vital roles in regulating intestinal health status and host energy metabolism, we detected the concentrations of butyric acid, propionic acid, and acetic acid in the feces. Our results showed that both the concentrations of acetate and butyrate for the high finishing weight group were significantly greater than the low finishing weight group (FDR-adjusted *p* < 0.05 and *p* < 0.01). No significant change was found in the levels of propionic acid between the two groups (Figure 6A). By further correlation analysis, we observed that the levels of acetic acid and butyric acid displayed a positive association with finishing weight, but the levels of propionic acid only exhibited a tendency of positive association with finishing weight (Figure 6B).

### Finishing weight associated species-SCFAs correlation

**Figure 7:**
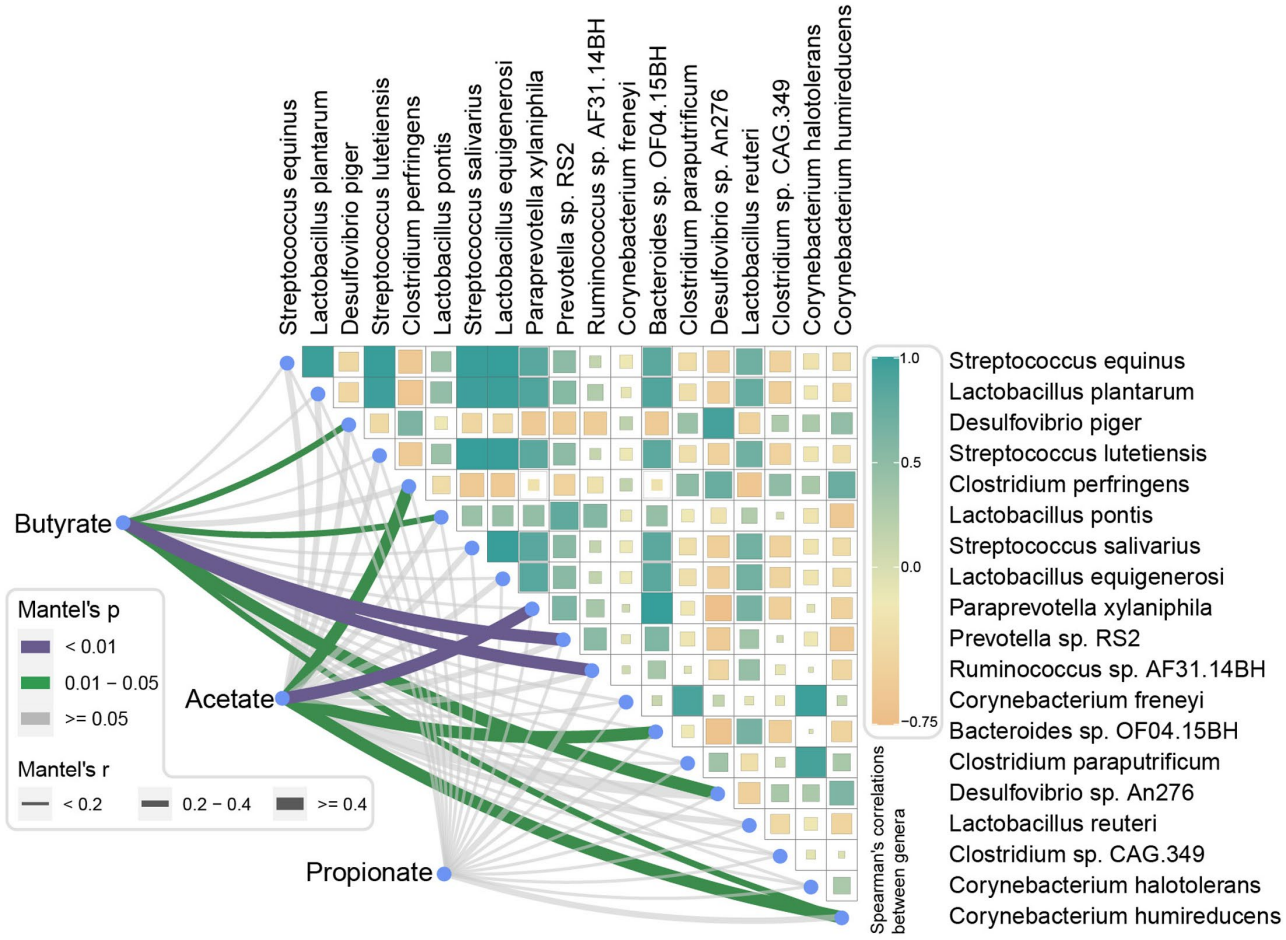
Correlations between short-chain fatty acid levels and finishing weight-associated species. Pairwise comparisons of finishing weight related-species are shown with a color gradient denoting Spearman’s correlation coefficient. Butyrate, acetate, and propionate were related to each microbial species by partial Spearman tests. Edge width corresponds to the Partial Spearman’s *r* statistic for the corresponding distance correlations, and edge color represents the statistical significance.

Mantel tests were performed to analyze the correlations between SCFAs level and finishing weight-associated species. As exhibited in Figure 7, we found that three species had positive relationships with the level of butyric acid, they were *Prevotella* sp. *RS2*, *Ruminococcus* sp. *AF31*-*14BH*, and *Lactobacillus pontis*, the species *Desulfovibrio* sp. *An276*, *Desulfovibrio piger*, and *Corynebacterium humireducens* showed negative correlations. Moreover, the level of acetic acid was positively related to *Paraprevotella xylaniphila* and *Bacteroides* sp. *OF04*.*15BH, respectively.* Nevertheless, no significant correlations were found between the level of propionic acid and finishing weight-associated species.

## Discussion

The pivotal roles of the gut microbiome in domestic animals have been continuously demonstrated, especially in aspects of nutrient metabolism, energy harvesting, and health maintenance (O'Callaghan et al., 2016). Accordingly, the gut microbiome is regarded as an important player in affecting the growth of livestock animals. Hence, identifying core gut microbiota is an important cut-in point for improving livestock production performances (Maltecca et al., 2020). SCFAs are essential metabolites generated by large intestinal anaerobic bacteria by fermenting the complex carbohydrates, which have emerged as an important modulator in gut health status and energy metabolism regulation (Koh et al., 2016). However, the effects of gut microbiota and SCFAs on the finishing weight of DSE pigs have received little attention. We, here, systematically and exhaustively assessed how the gut microbiome and SCFAs affect the finishing weight of DSE pigs.

A previous study indicated that overweight pigs have markedly lower species richness in alpha diversity and high dissimilarities in beta diversity (Panasevich et al., 2018). Another study also found that the higher body weight of Wuzhishan minipigs exhibited lower alpha diversity (Xu et al., 2022). We here confirmed these previously reported outcomes demonstrating that pigs with high body weight have lower gut microbial diversity and richness. In addition, in consistent with previous studies on the gut microbiota of swine (Long et al., 2021; Shang et al., 2022), we found that the gut microbiota of DSE pigs in both phylum and genus levels were dominated by *Firmicutes* and *Bacteroides*, which have a better capacity in producing various enzymes that are responsible for carbohydrate degradation and fermentation (Stojanov et al., 2020).

In total, 13 OTUs were specifically found in low and high finishing weight groups. Among these, four OTUs were detected in the high finishing weight group. *Prevotellaceae UCG*-*003* is a member of acetate-producing bacteria capable of degrading structural carbohydrates (cellulose and hemicellulose) in feeds and possesses beneficial roles in intestinal health by regulating the immune-inflammatory system (Li et al., 2019; Duan et al., 2021). Regarding the genus *Streptococcus*, which was subordinate to the order of *Lactobacillales* and the dominant lactate-producing genus in different segments of the intestine and also the main utilizers of simple carbohydrates (Chen et al., 2016). In contrast, *Desulfovibrionaceae* and *Corynebacterium* were identified in the low finishing weight group. Studies have shown that a high abundance of *Desulfovibrio* and *Corynebacterium* were detected in diarrhea-related cases (Zhu et al., 2018; Jo et al., 2021; Wang et al., 2021), suggesting that they might be the pathogenic bacteria that exert harmful effects on DSE pig’s growth and development.

Using metagenomic sequencing technology, we obtain more information about bacterial strains associated with finishing weight. A particularly notable finding is the larger proportion of *Streptococcus* strains in high finishing weight pigs, including *S*. *equinus*, *S*. *salivarius*, and *S*. *lutetiensis*. As the lactate-producing bacteria, *Bifidobacterium* and *Lactobacillus* are among the better described probiotics, the effect of *Streptococci* on the host intestine is less studied. A recent study demonstrated that *S*. *salivarius* exhibited an anti-inflammatory effect when co-incubated with gut epithelial cells (Li et al., 2022). Researchers found a remarkable increase of *S*. *lutetiensis* in the intestine after supplementing fructan (Vanhoutte et al., 2005). Furthermore, β-glucanases, α-galactosidase, and endoglucanases of *S*. *equinus* play a special role in degrading complex carbohydrates (Jans and Boleij, 2018). Commonly, *Streptococcus* mainly colonizes the surfaces of the intestinal mucosa. Studies provided supportive evidence that this genus could actively regulate peroxisome proliferator-activated receptors (PPARs; Are et al., 2008; Couvigny et al., 2015). These receptors, which were subordinate to the nuclear receptor superfamily of regulatory factors, are a class of ligand-activated transcription factors (Mirza et al., 2019). Their primary responsibility in the gut mucosa is to modulate genes related to lipid metabolism (Tenenbaum et al., 2003). They stimulate cell differentiation, especially as an essential inducer of adipocyte differentiation and growth (Brun et al., 1997; Lefterova and Lazar, 2009). Importantly, in human intestinal epithelial cells, *S*. *salivarius* has been demonstrated that significantly decreased the PPARγ-targeted metabolic genes (e.g., *I*-*FABP* and *Angptl4*), which were involved mainly in glucose metabolism and lipid accumulation (Couvigny et al., 2015). Consequently, down-regulating the level of PPARγ might lead to the effect of metabolic complications such as rapid weight gain. Here, we observe that the relative abundance of *Streptococcus* strains increased dramatically in the high finishing weight individuals. We hypothesize that the greater abundance of *Streptococcus* could fundamentally promote the enhancement of finishing weight by modulating energy harvesting and intake.

Furthermore, some species from *Lactobacillus* spp. could significantly affect finishing weight, such as *L*. *reuteri*, *L*. *plantarum*, *L*. *equigenerosi*, and *L*. *pontis*. In recent years, researchers unraveled the beneficial roles of *Lactobacillus* spp. on the growth performance of pigs. Supplementing extra *L*. *plantarum* in the pig diet could significantly increase both ADG and body weight (Yang et al., 2014). Another study has shown a significant elevation in feed conversion and ADG by dietary supplementation of *L*. *reuteri* (Hou et al., 2015). Additionally, the compound strains of *L*. *reuteri* and *L*. *plantarum* could decrease pig diarrhea rate (Dell'Anno et al., 2021). More importantly, the study pointed out that *Lactobacillus* spp. colonized the intestinal tract could elevate porcine growth hormone levels, antioxidation, and immunity, ultimately helping to fight stress (Yang et al., 2020). These investigations help explain why *Lactobacillus* spp. significantly enriched in high finishing weight pigs. We, therefore, speculated that *L*. *equigenerosi* and *L*. *pontis* may also serve as beneficial strains which play positive roles in pig growth performance.

We also observed that *Paraprevotella xylaniphila* was positively associated with finishing weight. A positive association between *P*. *xylaniphila* and obesity indices (body mass index, hip circumference, and waist circumference) for children who consumed high carbohydrate and saturated fat diets was found in a recent investigation (Orbe-Orihuela et al., 2022). Moreover, previous studies indicated that *P*. *xylaniphila* was a main succinate producing bacterium, it was positively associated with insulin, BMI, glucose, and triglycerides (Morotomi et al., 2009; Serena et al., 2018). On the contrary, certain species from *Clostridium* spp. and *Desulfovibrio* spp. showed negative correlations with finishing weight. *Clostridium perfringens* is a popular opportunistic pathogen inhabiting pig intestines (Zhou et al., 2021). The classical symptom of *C*. *perfringens* in pigs includes severe diarrhea, accompanied by intestinal villi damage and necrotic mucosa (Kiu and Hall, 2018). *Desulfovibrio piger*, which was a sulfate-reducing bacterium mainly degrades both sulfates and sulfites from the diet and generates hydrogen sulfide (H_2_S) as a product (Gibson, 1990). H_2_S can cause damage to the intestinal epithelium, triggering systemic and chronic inflammation (Attene-Ramos et al., 2007). More importantly, a newly published study indicated that *Desulfovibrio piger* was positively related to sarcopenia, which was typically characterized by weight loss (Wang et al., 2022). In short, these species augmenting the gut microbiome of DSE pigs could be the potential precipitating factors for the reduction of finishing weight.

The interaction network exhibited the co-exclusion and co-occurrence relationships among finishing weight-related species. Their interactions which could affect the finishing weight have a closer relationship with their diverse functions in host energy metabolism and intestinal health modulation. Similarly, earlier research indicated that the co-occurrence network embraced core genera including *Streptococcus*, *Lactobacillus*, *Bacteroides*, and *Prevotellaceae* were positively associated with porcine daily weight gains and body weight (Yang et al., 2021a). Moreover, the porcine body weight gain might be affected by the strong co-occurrence relationship between lactic acid producers (e.g., *Lactobacillus*) and butyrate-producer (e.g., *Ruminococcaceae*) through regulating host appetite and feeding behavior (Yang et al., 2018). Researchers also emphasized the essential role of gut microbial interactions in improving the growth and development of broiler chickens (Ma et al., 2018).

Compositions in metagenomic functional capacities were different between low and high finishing weight pigs. Both KOs and KEGG comparison analysis demonstrated that the glycolysis/gluconeogenesis, PTS, lipid metabolism, and secondary bile acid biosynthesis were more active in high finishing weight pigs, while the pathways of ABC transporters, sulfur metabolism, and one carbon pool by folate were overrepresented in low finishing weight group. The glycolysis/gluconeogenesis and PTS pathway were for the majority associated with being overweight (Graessler et al., 2013; Del Chierico et al., 2018). PTS exists only in bacteria where it gets involved in catalyzing the transportation and phosphorylation of carbohydrates (e.g., polysaccharides, monosaccharides, polyols, and amino sugars; Kashani et al., 2019). An earlier study indicated that both *Lactobacillus* and *Streptococcus* could ferment carbohydrates into lactate by their highly expressed PTS and carbohydrate metabolic genes (Shen et al., 2020). These two genera were overrepresented in high finishing weight pigs. This might be the main reason why these two pathways were active. Furthermore, the second bile acid (BA) biosynthesis, which is important for lipid and glucose metabolism regulation in the intestine (Zeng et al., 2019), was also active. Under the function of bile salt hydrolase (BSH), primary BA can undergo biotransformation to the secondary BA in the colon (Chiang, 2009). Intriguingly, previous studies have found that both *Lactobacillus* and *Streptococcus* were bile salt hydrolase (BSH) bacteria (Degirolamo et al., 2014), and *Streptococcus* was associated with lipid and bile acid homeostasis in the gut (Ren et al., 2017). It is possible that a high proportion of these two genera might contribute to increased concentrations of secondary BA and subsequently enhanced intestinal energy harvest and lipid metabolism to affect finishing weight. On the other hand, ABC transports are responsible for transporting various lipids, sterols, and drugs (Hou et al., 2017) and have recently been associated with multi-drug resistance and colonic inflammation (Andersen et al., 2015; Fang et al., 2017), which may be detrimental to growth performances in animals. Sulfur metabolism activated by sulfate-reducing bacteria (e.g., *Desulfovibrio*) has been demonstrated to be associated with inflammatory bowel diseases in mice (Metwaly et al., 2020). In addition, the biosynthesis pathway of folate seems to have a positive role in weight gain (Del Chierico et al., 2018). Recently, the research highlighted that one carbon pool by folate was enriched in Saba pigs with low body weight (Gao et al., 2020), which was in line with our study.

The CAZyme comparison analysis reflected that glucanase and glucosidase were more active in pigs with high finishing weights. Previous research has shown that glucanase supplementation could elevate the ratio of villi height to crypt depth, improve the absorption of nutrients, and finally promote body weight gain (Karunaratne et al., 2021). Furthermore, a greater abundance of glucosidase in the intestinal microbiome resulted in increased body weight gain in broilers (De Cesare et al., 2017). This could be used to explain why DSE pigs with a higher concentration of these two enzymes displayed greater finishing weight.

Our results also showed that both the levels of butyric and acetic acid were significantly increased in pigs with high finishing weights compared to pigs with low finishing weights, and these two SCFAs showed positive correlations with finishing weight. Butyrate possesses wide energy and metabolic modulation capacities and has strong anti-inflammatory effects that positively affect animal’s body weight gain (Bedford and Gong, 2018). On the other hand, research showed that an increased proportion of fecal and serum acetate contribute to the activation of the parasympathetic nervous system which reversely promotes elevated glucose-stimulated insulin secretion (GSIS), hyperphagia, and finally causes weight gain (Perry et al., 2016). Correlation analysis revealed positive associations between butyric level and *Prevotella* sp. *RS2*, *Ruminococcus* sp. *AF31-14BH*, and *Lactobacillus pontis*. As reported in previous literature, members from *Prevotella* possess butyrate kinase and phosphotransbutyrylase, which are essential for the biosynthesis of butyrate *via* the succinic pathway (Wang L. et al., 2020). Furthermore, *Ruminococcus* sp. *AF31*-*14BH* is a member that belongs to the family *Ruminococcaceae*, some species from it were well-known butyrate producers. However, it is necessary to perform future investigations for these two species and butyrate formation to support our findings. Additionally, research demonstrated a high abundance of intestinal *Lactobacillus* spp. contributed to the elevation of butyric acid levels (Le Roy et al., 2015), it makes sense to explain why there was a positive association between *Lactobacillus pontis* and butyric acid. Particularly, we observed that *P*. *xylaniphila* showed a positive association with acetic acids. According to the previous findings, *P*. *xylaniphila* was a bacterium that participated in glucose metabolism to produce acetic acids and succinic as the end products (Morotomi et al., 2009). Of note, evidence showed a strong association between levels of succinate/acetate and body weight (Serena et al., 2018; Sakamoto et al., 2022). Therefore, we suggested that *P*. *xylaniphila* might be a potential microbial marker for improving the growth performance of pigs.

Our study, although limited by a small pig population, offers important information that upholds the potential of intestinal flora manipulation in improving the finishing weight of DSE pigs. Nevertheless, future studies aiming to validate the positive or negative role of certain bacterial species in regulating finishing weight *via* specific pathogen-free (SPF) mice/minipigs’ intervention experiment and to obtain more mechanistic understandings into the cross-talk between gut microbes and host and its effects on growth performance of DSE pigs by multi-omics sequencing analysis are needed. In addition, supplementing the microbial species from *Lactobacillus* spp., *Streptococcus* spp., and *Prevotella* spp., or SCFAs (e.g., butyric and acetic acids) into the basal diet might be a valuable strategy to improve the finishing weight of DSE pigs.

## Conclusion

In summary, the present study detected some key microbial taxa related to finishing weight. We also performed functional annotation analysis and identified several finishing weight-associated CAZymes, KOs, and KEGG pathways. Furthermore, we highlighted the important role of SCFAs in finishing weight, especially acetate and butyrate. Hence, our investigation provides essential insights into the microbe-SCFAs interplay in affecting finishing weight and hints that modulating the intestinal bacterial communities could be an effective scheme to ameliorate finishing weight for DSE pigs and possibly other pig breeds.

## Data availability statement

The datasets presented in this study can be found in online repositories. The names of the repository/repositories and accession number(s) can be found at: NCBI BioProject—PRJNA910336.

## Ethics statement

The animal study was reviewed and approved by Animal Care and Use Committee (ACUC) in Hunan Agricultural University (Changsha, China; Permit Number: CACAHU 20210701).

## Author contributions

ML and YY conceived and designed the experiments, supervised the experiment progress, and revised the manuscript. YL, PP, LY, and HZ helped to collect the samples. QL performed the experiments, analyzed the data, and wrote the manuscript. PH, HM, and HW revised the manuscript. All authors contributed to the article and approved the submitted version.

## Funding

This study was supported by grants from the Natural Science Foundation of Hunan Province (No. 2022JJ40171), National Natural Science Foundation of China (No. 32002166), Breeding and Promotion of Lancang black pig (2021kjc-js072), Key Science and Technology Project of Yunnan Province (202202AE090032), Consulting research program of Yunnan Province pig industry development strategy (2022YNZH7-2), and the Science and technology innovation Program of Hunan Province (2021RC3091).

## Conflict of interest

The authors declare that the research was conducted in the absence of any commercial or financial relationships that could be construed as a potential conflict of interest.

## Publisher’s note

All claims expressed in this article are solely those of the authors and do not necessarily represent those of their affiliated organizations, or those of the publisher, the editors and the reviewers. Any product that may be evaluated in this article, or claim that may be made by its manufacturer, is not guaranteed or endorsed by the publisher.

## Supplementary material

SUPPLEMENTARY MATERIAL FIGURE S1 Finishing weight phenotypic values of all pigs (A) and low and high individuals (B). “**” represents FDR adjusted *p* < 0.01. “L” and “H” in the boxplot and PCoA plot represent low and high finishing weight, respectively.

SUPPLEMENTARY MATERIAL TABLE S1 Estimation of richness and diversity indices in gut microbiota among low and high finishing weight groups.

SUPPLEMENTARY MATERIAL TABLE S2 The relative abundances of OTUs in the phylum and genus level.

SUPPLEMENTARY MATERIAL TABLE S3 Differential microbial species between low and high finishing weight pigs.
