# Supplementary figures and images for: Association of gut microbiota and SCFAs with finishing weight of Diannan small ear pigs
Source: Front Microbiol. 2023 Jan 27;14:1117965. doi: 10.3389/fmicb.2023.1117965 (PMC9911695; doi:10.3389/fmicb.2023.1117965)

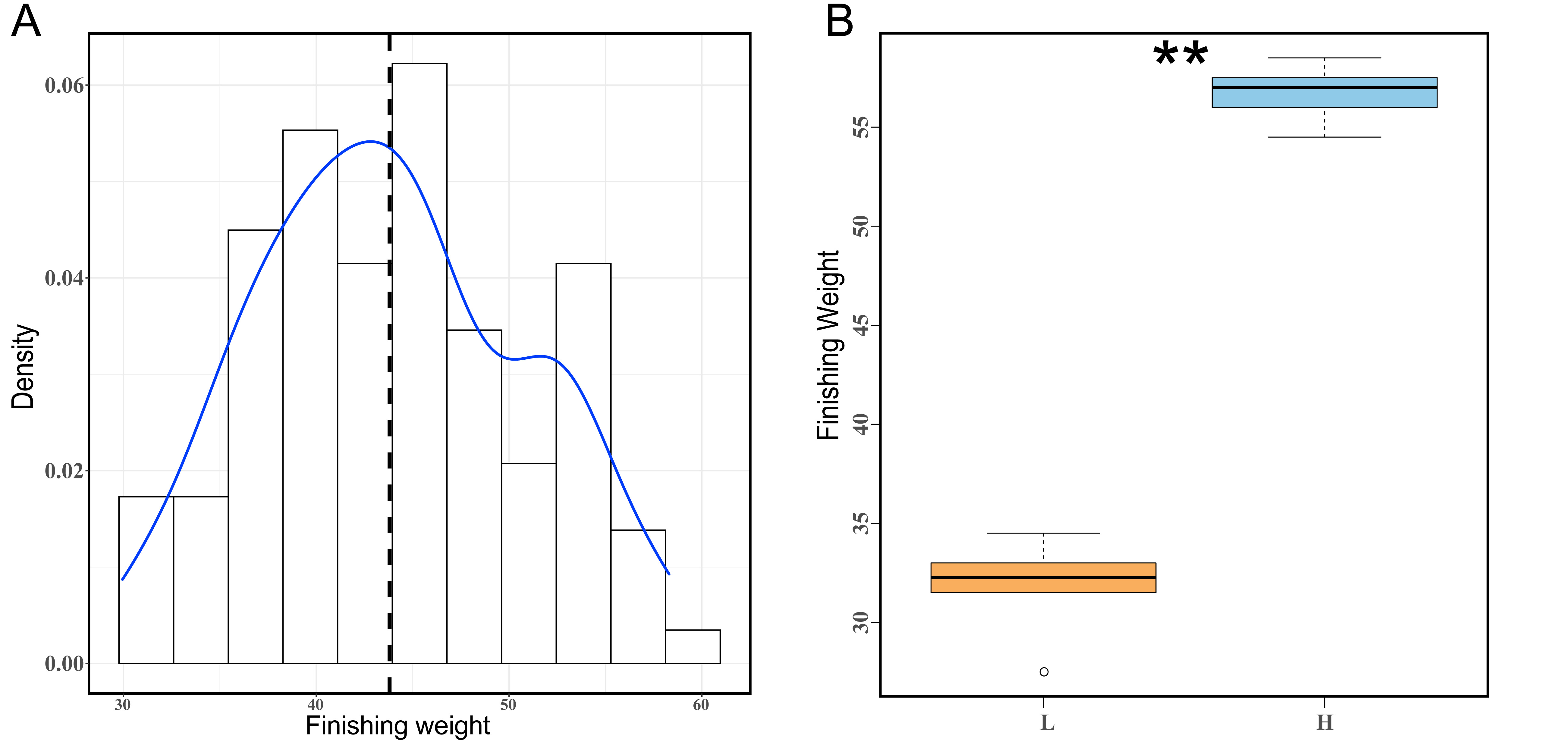

Supplement: SUPPLEMENTARY MATERIAL FIGURE S1 — Finishing weight phenotypic values of all pigs (A) and low and high individuals (B). “**” represents FDR adjusted p < 0.01. “L” and “H” in the boxplot and PCoA plot represent low and high finishing weight, respectively. [file Data_Sheet_1.zip › Figure. S1.jpg]
